# Disability and Pain are the Best Predictors of Sick Leave After a Distal Radius Fracture in Men

**DOI:** 10.1007/s10926-020-09880-4

**Published:** 2020-02-12

**Authors:** Lisa Egund, Karin Önnby, Fiona Mcguigan, Kristina Åkesson

**Affiliations:** 1 Department of Clinical Sciences Malmö, Clinical and Molecular Osteoporosis Research Unit, Lund University, Lund, Sweden; 2 Department of Orthopedics, Skåne University Hospital, 205 02 Malmö, Sweden

**Keywords:** Distal radius fracture, Men, Sick leave, Outcome, Disability, Pain

## Abstract

*Purpose* Distal radius fracture often compromises working ability, but clinical implications are less studied in men due to its lower incidence. This study therefore describes sick leave in men with distal radius fracture, specifically exploring the impact of patient- and fracture-related factors. *Methods* Professionally active men aged 20–65 with distal radius fracture were followed prospectively for 1-year (n = 88). Data included treatment method, radiographic parameters pre/post treatment, complications, health, lifestyle and occupational demand. Patient outcomes were self-reported sick leave; Disability of the Arm, Shoulder and Hand (DASH) score; pain (5 likert scale); SF-36: Physical Component Scale (PCS) and Mental Component Scale (MCS). *Results* Median sick leave was 4 weeks (IQR 0; 8); almost a third reported taking no sick leave. Categorizing sick leave into 3 groups (0–6, 7–12 and > 12 weeks), men with the longest sick leave had 22 points higher DASH score (p = 0.001) and 5 points lower PCS (p = 0.02) at 1 week and the difference remained over time; they were also older and more often treated surgically. The strongest predictors of length of sick leave were one-week post-fracture DASH score (rs = 0.4, p < 0.001), pain intensity (rs = 0.4, p < 0.001) and PCS (rs = − 0.4, p = 0.002). The correlation between sick leave and pain was even stronger analyzing treatment groups separately (closed reduction and cast r_s_ = 0.56, p = 0.007, surgery r_s_ = 0.42, p = 0.04). *Conclusions* Self-reported disability, pain and global health measurements as early as 1 week post-fracture are the strongest predictors of length of sick leave regardless of treatment; an important finding easily transferrable to clinical management of distal radius fractures.

## Introduction

The most frequent fracture in adults is the distal radius fracture. The reported incidence rate in Southern Sweden is as high as 278 per 100,000 person years and increasing, especially among those of working age [1, 2]. Although a distal radius fracture is often regarded as simple with good prognosis, a considerable number of individuals experience prolonged pain and disability [3].

Time lost from work is a common negative consequence after a fracture. Trauma related incapacity is an issue clinicians deal with every day, although not much studied. Specific factors predictive of length of post-fracture sick leave are still poorly understood; for distal radius fracture only one study has specifically addressed this question [4]. Since men comprise a minority of this fracture population, determinants of sick leave are even less evaluated. From a socio-economic view point, and in the light of an increasing incidence, it is essential to recognize the impact and cost for employers and individuals, as well as where to intervene to reduce this burden.

A commonly held belief is that high work demand and more severe fracture lead to prolonged sick leave [5, 6], whereas we hypothesize that patient perception also plays a key role. The aim of the present study is to describe the variation in sick leave among men with distal radius fracture and most importantly to explore the impact of patient-related factors such as self-reported disability and pain, in addition to fracture related variables.

## Methods

### Participants and Design

We conducted a cohort study of men with distal radius fracture at the Department of Orthopedics, Skåne University Hospital, Malmoe. The main focus was bone mass and risk factors of osteoporosis as earlier described [7]. As part of this larger study, a prospective study was designed to follow patients during 12 months post-fracture and in addition to bone parameters also evaluating functional outcome and sick leave. Consecutive patients with an acute distal radius fracture were invited during 2003–2007. General inclusion criteria: male, age 18–65 years, in active employment or education, acute distal radius fracture and resident in the catchment area. Exclusion criteria were multiple fractures (including bilateral radius fracture), cognitive impairment and insufficiently understanding Swedish to complete the questionnaires.

All parts of the study were approved in advance by the Lund University ethical review board (November 11th 2002, LU 788 02) and performed in compliance with the Helsinki Declaration. All participants gave written informed consent.

We initially identified 333 men aged 18–65 years with distal radius fracture, of whom 89 did not meet the inclusion criteria (36 with non-acute fracture; 20 with multiple fractures; 10 non-residents, 13 non-Swedish speaking; 10 retired or not in active employment/education). Of the 244 eligible, 88 agreed to participate. Reasons for non-participation were general unwillingness (117), illness (18), known substance abuse (14) non-contactable (7). Age distribution of participants and non-participants did not differ.

### Treatment Protocol

Participants were all treated according to established standard treatment protocol for distal radius fracture [8]. Undisplaced or minimally displaced fractures were treated in a short arm cast for 4–5 weeks, unstable fractures were treated with closed reduction and cast and highly unstable fractures were surgically treated (mainly external fixation at the time of investigation). The patients were seen by a physiotherapist within one week after the cast or external fixator removal and again at a final routine check 3–4 weeks later.

### Data Collection

At enrollment, the participants completed a comprehensive questionnaire on health status, medication and life-style factors. Trauma level was recorded—low trauma defined as a fall from standing height or less; high trauma as all other types. Fracture complications within the first year post-fracture were identified retrospectively by reviewing patient records. As a measure of pre-existing comorbidity we calculated Charlson Comorbidity Index (CCI) from the information available in the initial questionnaire.

Socio-economic status (educational level, type of work, workload) was recorded and work demand classified as sedentary, light, medium, heavy, very heavy [9]. Sick leave was self-reported and recorded by questionnaire at 6–8 weeks, 3, 6 and 12 months. In Sweden, the national health insurance allows compensation up to one year when unable to work due to sickness or injury.

### Assessment of Disability and Global Health

The Disability of the Arm, Shoulder and Hand questionnaire (DASH, Swedish version) was recorded at inclusion approximately one week after fracture, and again at 6–8 weeks and 12 months. DASH is a 30 item instrument evaluating disability of the upper limb, which provides an overall score of 0–100; higher scores indicating higher disability [10]. In addition, in order to further understand the importance of patient perceived pain on sick leave we have specifically analyzed the pain question within the instrument.

As measurements of global health, participants completed the SF-36 questionnaire (1 week; 6–8 weeks; 12 months) and the EQ-5D-3L (6–8 weeks; 12 months) after fracture.

The SF-36 health status questionnaire has 36 items, producing eight scales in various aspects of physical and mental health, compressible into two overall scores: the physical component score (PCS) and the mental component score (MCS). Each is normalized to a mean of 50 and standard deviation of 10 compared to the general US population, with higher score indicating better quality of life [11].

The EQ-5D-3L consists of the EQ-5D descriptive system and the EQ visual analogue scale, EQ VAS. The latter records the respondent’s self-rated health on a vertical, visual analogue scale where the endpoints are labelled ‘Best imaginable Health state’ and ‘Worst imaginable Health state’ [12].

### Radiographic Evaluation

Standard postero-anterior and lateral radiographs were obtained at admission to the emergency department and at the routine follow-up 7–10 days later. We evaluated the initial and the latest available radiographic examination measuring dorsal tilt (degrees) and ulnar variance (mm). Dorsal tilt was measured on the lateral view as the angle between a line connecting the dorsal and volar lips of the distal radius and a line perpendicular to the central axis of the radius [13]. Ulnar variance was measured on the posteroanterior view by the method of perpendiculars and central reference point [14, 15]. Radiographs were assessed using Sectra IDS7 version 18.2.18.4066 (Sectra AB, Linkoping Sweden).

### Statistics

Categorical variables are expressed as numbers and continuous variables as mean with standard deviation (SD) and/or range. We used independent unpaired t-test for continuous variables and chi-square for comparisons between categorical variables. DASH data are ordinal with a skewed distribution, therefore non‐parametric analyses were employed, and data presented as median (interquartile range, IQR).

Sick leave was analyzed in three clinically relevant groups: ‘short’ 0–6 weeks (immobilization and two weeks rehabilitation), ‘intermediate’ 7–12 weeks (allowing for longer rehabilitation) and ‘prolonged sick leave’ ≥ 13 weeks. Work demand was compressed into two categories due to the relatively small cohort size: sedentary-medium and heavy-very heavy [16].

To explore associations between specific variables and sick leave category, ANOVA was used for normally distributed, Kruskal–Wallis for non-normally distributed continuous variables and Chi-square for categorical variables. Spearman’s rho (r_s_) was used for bivariate correlation analysis (which allows inclusion of outliers, non-normally distributed or non-linearly related variables) and is presented with 95% confidence interval.

Prior to regression analysis, diagnostics identified three outliers (each with 42 weeks or more of sick leave) which were excluded; residuals from each analysis were thereafter normally distributed. The multiple regression analysis, included age, CCI and work demand (Model 1) i.e. non-modifiable, non-fracture related factors. Model 2 included age, CCI, work demand and fracture treatment (cast, closed reduction and cast or surgery). To each of these models, disability, pain, or global health were added as explanatory factors. Data is presented as R^2^ and unstandardized regression coefficient (B), with 95% confidence interval.

Analyses were performed using SPSS version 25 (IBM Corp., NY, USA). We considered a two-tailed p-value < 0.05 statistically significant.

## Results

Mean age at fracture was 45 (21–64), (median 48, IQR 33; 57). Twenty-two patients sustained their facture while at work, 25 at sport activities and the remaining at other leisure activities. Almost half of the reported fractures resulted from high energy trauma (Table 1).

**Table 1:** Patient- and fracture related factors and disability/global health of all men of working age with distal radius fracture and also by duration of sick leave

	All DRF Patients	Short 0–6 weeks	Intermediate 7–12 weeks	Prolonged > 12 weeks	*p*-value^§^
	N = 88	N = 56	N = 25	N = 7	
*A) Patient related factors*					
Age, years (SD)	45 (14)	43 (14)	49 (13)	53 (10)	0.038^¶^
BMI, kg/cm^2^ (SD)	26.1 (3.8)	25.3 (4.0)	27.5 (3.4)	27.1 (2.3)	0.065^#^
Charlson Comorbidity Index (SD)	0.3 (0.6)	0.3 (0.6)	0.3 (0.6)	0.9 (1.1)	0.006^††^
Alkohol, units (10 g)/week (SD)	9 (8)	10 (8)	8 (9)	6 (3)	0.245^¶^
Work status					
Professionally active	82	50	25	7	
Student	6	6	0	0	
Educational level					
Primary	17	9	5	3	0.089^††^
Secondary	38	21	13	4	
University	28	23	5	0	
Work demand					
Sedentary–medium	72	50	17	5	0.066^††^
Heavy–very heavy	16	6	8	2	
*B) Fracture related factors*					
Trauma level high	38	24	12	2	0.016^††^
Dominant hand fracture^a^	33	23	7	3	0.487^††^
AO classification					
A	19	12	4	3	0.105
B	11	10	0	1	
C	50	29	18	3	
Displacement^β^—initial	29/75	14/47	12/22	3/6	0.121^††^
Displacement-follow up	4/77	2/50	2/21	0/6	0.529^††^
Treatment					
Cast	42	33	7	2	0.024^††^
Closed reduction + cast	22	14	6	2	
Surgery	24	9	12	3	
Complication-major	4	1	1	2	0.023^††^
*C) Disability/global health*					
DASH (median (IQR))					
1 week	41 (29;57)	34 (23;47)	50 (38;59)	56 (48;60)	0 001^¶^
6–8 weeks	14 (6;27)	10 (4;18)	23 (7;31)	27 (13;31)	0 011^¶^
12 months	2 (0;7)	1 (0;3)	6 (1;12)	9 (8;28)	< 0.001^¶^
SF-36 [mean (SD)]					
1 week					
PCS	40 (6)	41 (6)	37 (6)	36 (3)	0.016^#^
MCS	48 (11)	47 (11)	49 (12)	46 (8)	0.811^#^
6–8 weeks					
PCS	43 (8)	45 (7)	40 (8)	38 (5)	0.004^#^
MCS	52 (10)	52 (9)	52 (13)	48 (9)	0.582^#^
12 months					
PCS	52 (7)	54 (6)	51 (8)	43 (6)	0.001^#^
MCS	53 (6)	53 (5)	51 (9)	54 (8)	0.418^#^
EQ5D VAS 6–8 weeks (mean ± SD)	80 (13)	83 (10)	77 (14)	68 (23)	0.008^#^

Data is presented as numbers unless otherwise stated and vary slightly due to missing data

^a^n = 17 missing information. ^β^Dorsal tilt > 10º and/or ulnar variance > 2 mm

^§^*p*-value for observed differences between the 3 sick leave groups. Statistical methods: ^¶^Kruskal–Wallis

^#^One-way ANOVA

^††^Chi-Square

**Fig. 1:**
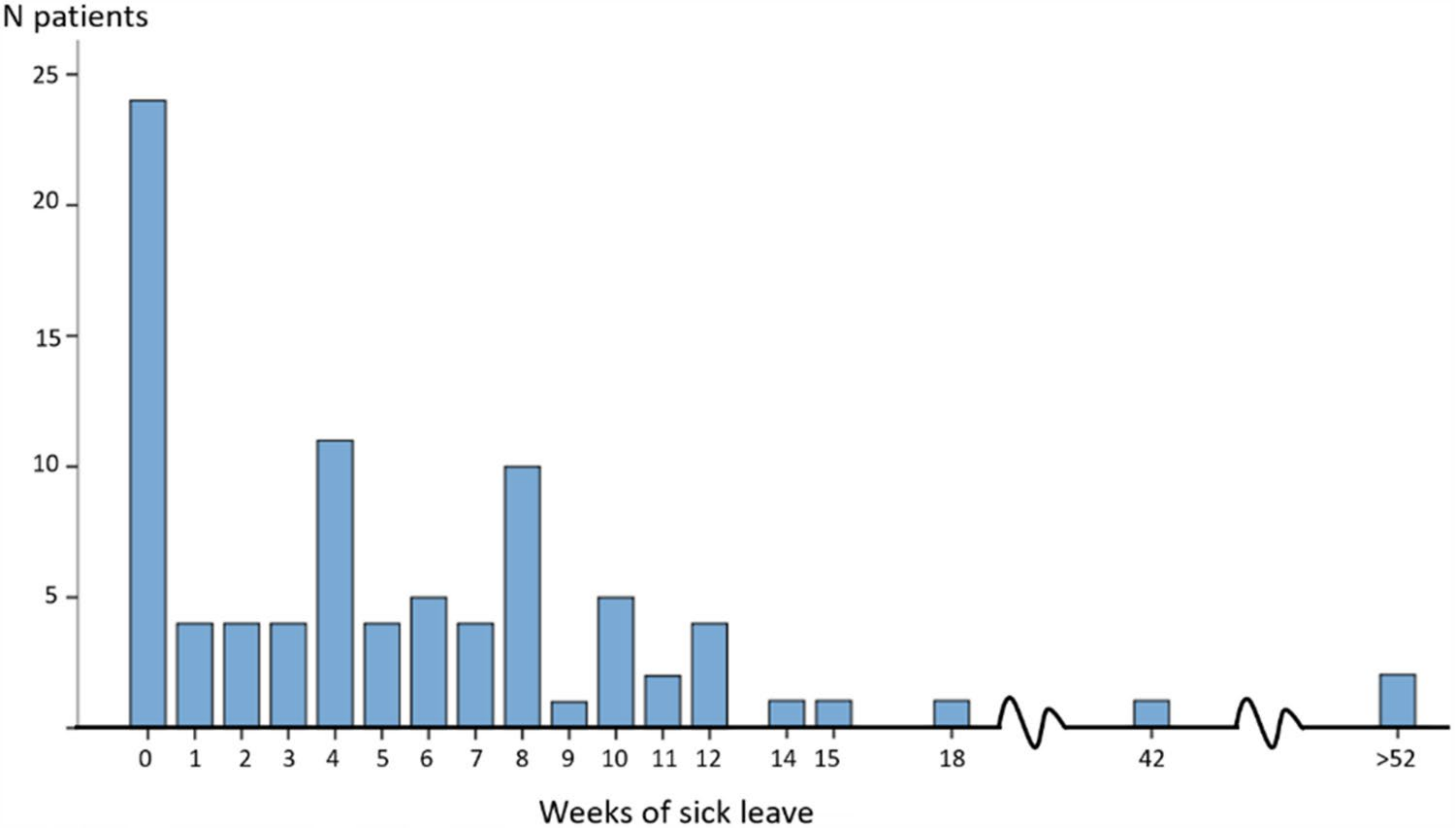
Distribution of weeks of sick leave after distal radius fracture in men

Figure 1 shows the distribution of sick leave among the participants which ranged from 0 to 52 weeks (median 4; (IQR 0; 8); mean 6 SD 9). Almost one third (24/88) did not require any sick leave; these men were younger (p = 0.02) and had a higher education level (p = 0.002). Length of sick leave also differed between non-surgical or surgical treatment (median 4 vs 8, p = 0.008). Two had major complications and had not returned to work by the conclusion of the study (one tendon rupture, one displacement requiring surgery within the first year); no cases of Complex Regional Pain Syndrome (CRPS) were reported.

### Sick Leave Groups

To evaluate patient and fracture related factors which might be associated with sick leave, we used three sick leave groups: short, intermediate and prolonged. Men in the prolonged sick-leave group were more likely to have had surgery compared to men with shorter sick leave (Table 1), although timing of surgery had no impact; there was no difference between those who had primary surgery compared to those having secondary surgery due to displacement at the weekly follow-up. There was no difference in length of sick leave in those having AO type A (7 weeks), B (6 weeks) or C (6 weeks) fracture (p = 0.959). Men with the longest sick leave tended to be older with low trauma fractures being more frequent.

**Fig. 2:**
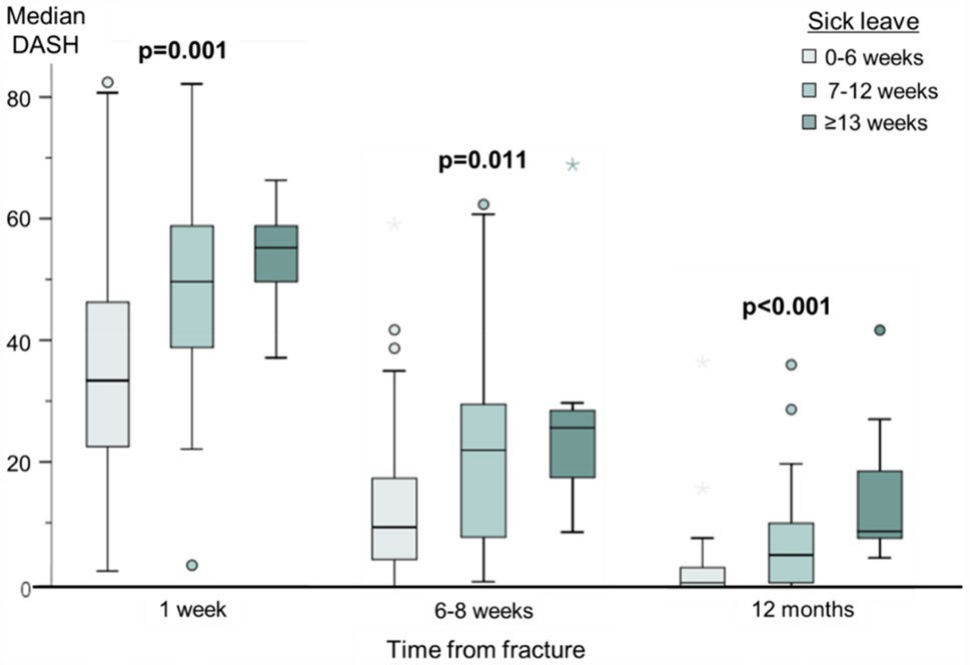
Self-reported disability (DASH score) at 1 week, 6–8 weeks and 12 months by sick leave category. Reported values are median DASH score and 95% Confidence Intervals

**Fig. 3:**
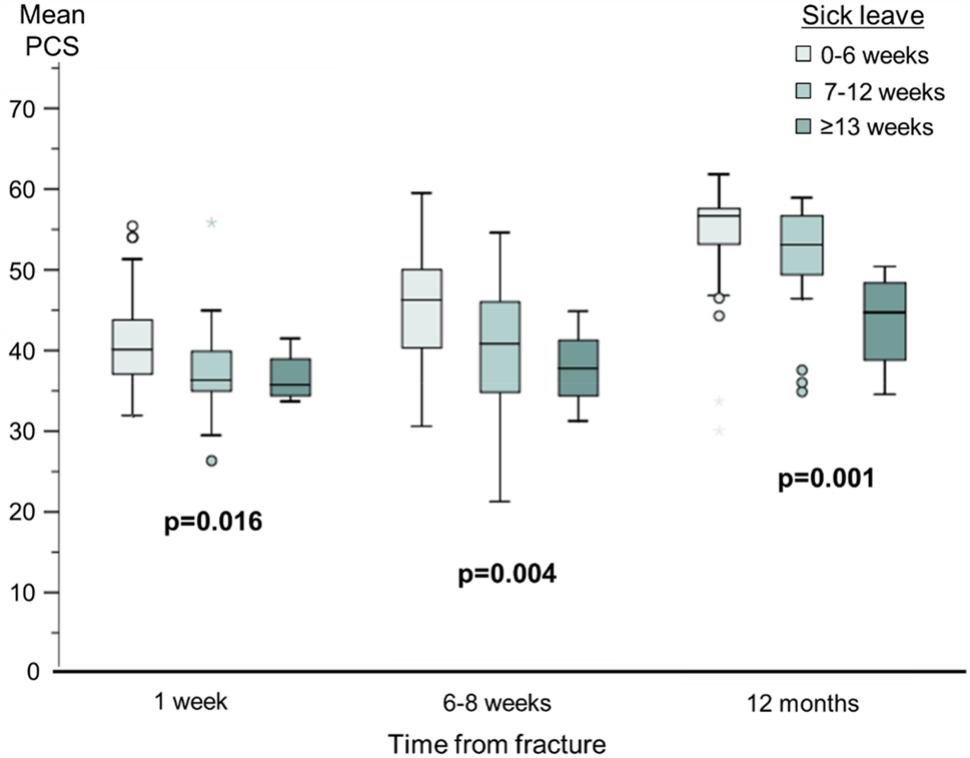
Self-reported global health (SF-36 PCS) at 1 week, 6–8 weeks and 12 months by sick leave category. Reported values are mean SF-36 Physical Component Score (PCS) and 95% Confidence Intervals

Overall, men with the longest sick leave had higher DASH scores and lower PCS at all time-points, although all three groups improved over time. The DASH score at 1 week was 22 points higher in those with prolonged sick leave compared to those with short (p = 0.001) (Table 1, Fig. 2) and PCS was significantly lower (Table 1, Fig. 3). The difference in DASH score and PCS remained at 12 months post-fracture, most pronounced for PCS. Men with intermediate sick leave and even more pronounced in the prolonged group showed significantly lower EQ5D VAS scores compared to those in the short (p = 0.008)(Table 1).

### Predictors of Length of Sick Leave

Correlations with length of sick leave were then explored; consistent associations were observed with self-reported disability and general health (Table 2). The correlation between length of sick leave and DASH score, intensity of pain and PCS was apparent already at one week after fracture. The correlation between sick leave and pain at one week was even stronger when analyzing treatment groups separately (closed reduction and cast r_s_ = 0.56, p = 0.007, surgery r_s_ = 0.42, p = 0.04). There was no correlation between global mental health MCS and sick leave at any time point.

**Table 2:** Correlations, r_s_ with 95% CI, between duration of sick leave and self-reported disability/global health and patient/fracture related factors

		Spearmans r_s_ (95% CI)	*p*-value
*A) Disability/global health*			
1 week			
DASH score	0.38	(0.19, 0.55)	< 0.001
DASH Pain	0.41	(0.22, 0.57)	< 0.001
SF-36 PCS^a^	− 0.35	(− 0.53, − 0.14)	0.002
SF-36 MCS^b^	− 0.05	(− 0.27, 0.18)	0.687
6–8 weeks			
DASH score	0.38	(0.17, 0.56)	0.001
DASH Pain	0.37	(0.16, 0.55)	0.001
SF-36 PCS	− 0.41	(− 0.58, − 0.21)	< 0.001
SF-36 MCS	− 0.08	(− 0.39, 0.15)	0.516
EQ5D VAS	− 0.30	(− 0.50, − 0.075)	0.011
12 months			
DASH score	0.52	(0.32, 0.67)	< 0.001
DASH Pain	0.30	(0.075, 0.50)	0.010
SF-36 PCS	− 0.46	(− 0.63, − 0.25)	< 0.001
SF-36 MCS	0.03	(− 0.21, 0.27)	0.785
EQ5D VAS	− 0.34	(− 0.53, − 0.12)	0.004
*B) Patient related factors*			
Age	0.31	(0.11, 0.49)	0.004
CCI	0.03	(− 0.18, 0.24)	0.779
Alcohol, units/week	− 0.14	(− 0.35, 0.084)	0.237
Educational level	− 0.36	(− 0.53, − 0.16)	0.001
Work demand^c^	0.28	(0.071, 0.47)	0.008
*C) Fracture related factors*			
Trauma level	0.0003	(− 0.21, 0.21)	0.974
Dominant hand fracture	− 0.21	(− 0.42, 0.018)	0.077
Displacement^d^ initial	0.27	(0.046, 0.47)	0.021
Displacement^d^ follow-up	0.028	(− 0.20, 0.25)	0.809
Treatment^e^	0.29	(0.086, 0.47)	0.006
Complication-major	0.078	(− 0.13, 0.28)	0.471

^a^Physical Component Scale

^b^Mental Component Scale

^c^Dichotomized into light-medium/high-very high

^d^Dorsal tilt > 10° and/or ulnar variance > 2 mm

^e^Treatment categories: cast, closed reduction and cast, surgery

Conventionally evaluated factors e.g. treatment method and radiographic measurements generally showed weaker association to length of sick leave (Table 2). Age was correlated to sick leave as well as educational level, although the effect of the latter was diminished when adjusting for work demand.

Fracture severity at the initial presentation, measured as radiographic displacement (dorsal tilt > 10° and/or ulnar variance > 2 mm) and treatment type were associated with sick leave. Of the radiographic parameters only dorsal tilt at *initial* presentation correlated with sick leave (r_s_ =  − 0.3, p = 0.02). Ulnar variance or dorsal tilt at *follow-up* evaluation were not associated. Neither trauma level nor involvement of the dominant hand correlated with length of sick leave.

Finally, to identify potential predictors of length of sick leave, we performed stepwise multiple linear regression analysis (Table 3). The ground model, which included non-fracture related factors i.e. age, CCI and work demand, explained 19% of the variation in length of sick leave. The addition of disability, intensity of pain or perception of global health at either one or 6–8 weeks after fracture to this model increased prediction to 23–27%.

**Table 3:** Predictors of length sick leave after distal radius fracture

	*R*^*2*^	B [95% CI]	*R*^*2*^	B [95% CI]
Model 1				
Age	0.19	0.12 [0.043, 0.19]		
CCI^a^		− 0.60 [− 2.11, 0.94]		
Work demand^b^		3.96 [1.56, 6.37]		
	1 week		6–8 weeks	
DASH score	0.25	0.050 [0.003, 0.096]	0.23	0.075 [0.005, 0.15]
DASH Pain	0.27	1.18 [0.15, 2.22]	0.25	1.12 [0.083, 2.27]
SF-36 PCS	0.23	− 0.12 [− 0.28, 0.031]	0.27	− 0.20 [− 0.33, − 0.070]
Model 2				
Age	0.28	0.086 [0.018, 0.15]		
CCI		− 0.68 [− 2.09, 0.73]		
Work demand		4.01 [1.76, 6.27]		
Treatment^c^		1.47 [0.43, 2.51]		
	1 week		6–8 weeks	
DASH score	0.32	0.036 [-0.010, 0.082]	0.34	0.062 [− 0.006, 0.13]
DASH Pain	0.35	0.96 [− 0.035, 1.96]	0.35	1.036 [− 0.020, 2.09]
SF-36 PCS	0.32	− 0.095 [− 0.25, 0.057]	0.37	− 0.21 [− 0.33, − 0.085]

The stepwise multiple regression relies on the ground model including age, CCI† and work demand‡ (model 1) or with treatment (model 2) and separately adding self-reported disability, pain or physical health. Data are presented as R^2^ and unstandardized regressions coefficient, B, with 95% CI

^a^Charleson Comorbidity Index

^b^Dichotomized into light-medium/high-very high

^c^Treatment in three groups: cast, closed reduction and cast, surgery

Recognizing that fracture severity and hence type of treatment influence rehabilitation, model 2 included treatment method which then explained 28% of the variation in sick leave. The addition of self-reported measures of disability and global health i.e. 1-week DASH score or intensity of pain added significantly to prediction, now explaining 32% and 35% of sick leave, increasing to 35% and 37%) at 6–8 weeks.

## Discussion

In the present study, we find that sick leave after a distal radius fracture in men of working age is highly variable. Almost one quarter of men with radius fracture do not lose any time from work and few are unable to return to work 12 months after fracture. The most consistent finding was the impact of self-reported disability and physical health on length of sick leave and most interestingly, the early prediction of prolonged sick leave by the intensity of pain.

The only previous study on the subject is by Macdermid et al. [4] and we find both differences and similarities. The cohorts are comparable regarding age, dominant hand fracture, prevalence of surgery and work demand,however, although accounting for forty percent of their cohort, men were not analyzed separately. There is a quite large difference in length of sick leave following distal radius fracture between the studies; Macdermid found a median 8 weeks sick leave compared to only 4 weeks in our male only cohort. Whether this finding is being skewed by including women or if there is an impact of differences in social insurance systems is difficult to judge. Participants in the Macdermid study exhibited much worse DASH scores at three and twelve months, although the work loss categories are not quite the same and notably, it is unclear whether median or mean DASH scores are reported. However, both studies share the main conclusions: that approximately twenty percent do not lose any time off work and that self-reported disability is the strongest predictor of length of sick leave.

A finding of greater importance is that higher disability and pain intensity as early as 1 week after a distal radius fracture, is a risk factor for longer sick leave in men, whether treated non-surgically or surgically. This is supported by an earlier study, showing that baseline pain intensity predicts chronic pain in both men and women with distal radius fracture [17].

The subjective symptom “pain” is multifactorial involving both injury (fracture severity, treatment, complications) and patient related factors (age, educational level, prior pain experience and psychosocial factors) [18]. Many of these factors are nonmodifiable but should be considered,early identification of risk factors leading to chronic musculoskeletal pain following an acute injury may minimize the risk of persistent pain, disability and prolonged sick leave. There is still no consensus on the most appropriate rehabilitation program, supervised therapy versus a home exercise program, following a distal radius fracture in adults [19] and current recommendation emphasize clinicians to rely on their experience and clinical judgement [20]. Our study indicates, that a higher degree of pain at an early stage, regardless of treatment type, is predictive of longer sick leave and as a yellow flag assist the clinician in allocating rehabilitation resources and other pain-reducing treatment.

We found that higher age at fracture was associated with longer sick leave which contrasts with the Macdermid study [4]. Lower education is a known risk factor for unhealthy life style and sick leave [21, 22]. In the present study, however, the association between sick leave and educational level was diminished when adjusting for work demand since work demand was higher in those with lower education. Remarkably, we didn’t find the strong association between work demand and sick leave as would be expected.

On the other hand, we find that aspects traditionally regarded as important, such as fracture of the dominant hand, fracture severity measured by classification and displacement at follow-up, were not associated with longer sick leave. However, only a few had displacement at follow-up, which might preclude statistical significance. High trauma level in the non-osteoporotic population has been hypothesized to correlate to more severe soft tissue injury [23],we did not find any correlation between trauma level and sick leave. At the time of this investigation surgery was primarily external fixation for 5 weeks, while presently the current trend favors volar plates as the treatment of choice, which may result in shorter length of sick leave due to earlier mobilization [24].

The main strengths of this study are the prospective design, the continuous follow-up and most importantly, the explicit focus on men, who have been excluded from or comprised a minority in many prior studies. In contrast to most of the current research which concentrates on fracture severity and treatment methods, we focus on self-reported outcome measurements in addition to traditionally evaluated factors providing further insight regarding sick leave after distal radius fracture in men. It also becomes clear that larger clinical investigations are warranted to address the increasing prevalence of distal radius fracture in the expanding working-age population and to improve identification of individuals at risk of long sick leave.

We recognize several limitations. Firstly, the cohort is relatively small and hence may be underpowered to detect differences especially in those groups with low numbers, i.e. participants with prolonged sick leave and displacement at follow-up. There is also the risk of selection bias; however, we found no difference in age distribution between participants and non-participants. This cohort is part of a study on osteoporosis in men with distal radius fracture. Our participation rate is similar to equivalent osteoporosis studies, but different from mail-based studies focusing only on self-reported outcome [25, 26] with physical participation being more demanding than returning questionnaires. Furthermore, studies in men often have lower participation rates [25–27]. Thirdly, we did not have objective functional measurements such as range of motion or grip strength, which could have been valuable. On the other hand, these variables have not been shown to clearly correlate with disability in terms of DASH scores [28]. Fourthly, our sick leave data was self-reported, which may not fully agree with administrative data [29, 30]. Recall bias can be expected, but we believe that the continuous follow-up reduces such impact.

Finally, we recognize the complexity of sick leave after a fracture. Although this study was based on prospectively collected data, the research question was designed after conclusion of the study. Thus, not all potentially interesting predictors were included (work culture, social situation and the individual’s sense of coherence). However, this was not within the scope of this study and we focus on factors readily available in daily clinical management, aware that we are missing important pieces of the puzzle.

We conclude that for men of working age with distal radius fracture, although duration of sick leave is highly variable, those with higher perceived disability and pain as early as 1 week post-fracture have longer sick leave, a finding that should influence clinical management directly. Furthermore, patient-reported measures are more predictive of length of sick leave than traditional clinical variables such as radiographic presentation and should be incorporated in the standard management.
